# Variation between nematodes in a multi-sensory behavioral assay

**DOI:** 10.17912/micropub.biology.000330

**Published:** 2020-11-28

**Authors:** Renae Ellis, Gareth Harris

**Affiliations:** 1 Biology Program, 1 University Drive, California State University Channel Islands, Camarillo, Ca, 93012

**Figure 1. Variation between nematodes in a multi-sensory behavioral assay: Different nematode species show distinct food leaving rates during exposure to Repulsive cues:**
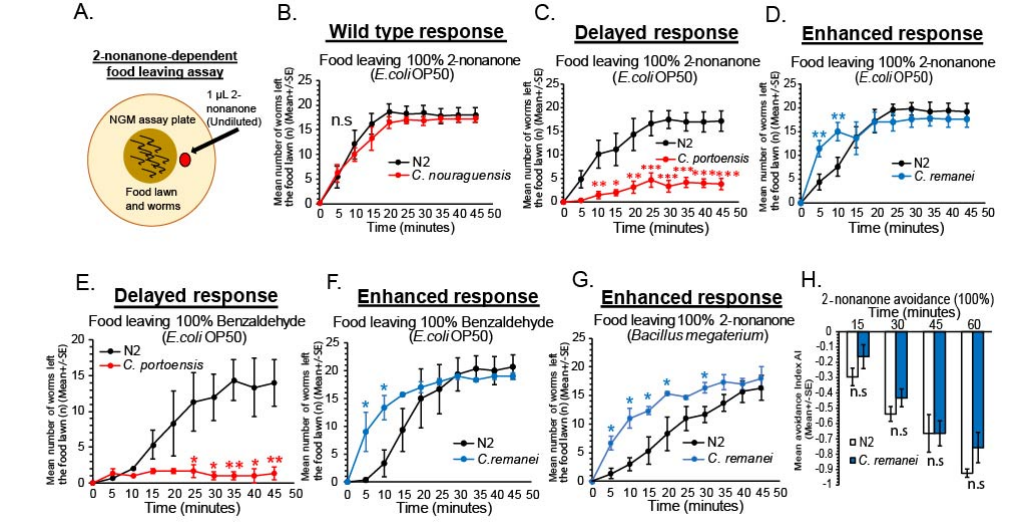
**A-H** **A)** Schematic of behavioral assay used to examine 2-nonanone-dependent food leaving (See methods section), **B)** Wild type N2 *Caenorbabditis elegans* vs *Caenorhabditis nouraguensis* examined for 2-nonanone-dependent food leaving, (n=5). Wild type N2 *C. elegans* (Black line), *C. nouraguensis, (*Red line), n.s=not statistically different, **C)** Wild type N2 *Caenorhabditis elegans* and *Caenorhabditis portoensis* were examined for 2-nonanone-dependent food leaving (n=6). Wild type N2 *C. elegans,* (Black line), *C. portoensis,* (Red line), **D)** Wild type N2 *Caenorhabditis elegans* vs *Caenorhabditis remanei* were examined for 2-nonanone-dependent food leaving (n=6). Wild type N2 *C. elegans,* (Black line), *C. remanei,* (Blue line), **E-F)** *Caenorhabditis portoensis* (slow leavers) and *Caenorhabditis remanei* (fast leavers) show same food leaving behavior in response to a second repellent, undiluted benzaldehyde (n=3 and, n=4, respectively). Wild type N2 *C. elegans*, (Black line), *C. portoensis*, (Red line), and wild type N2 *C. elegans*, (Black line), *C. remanei*, (Blue line), **G)** *Caenorhabditis remanei* shows fast food leaving behavior during exposure to 2-nonanone while residing on a *Bacillus megaterium* food patch (n=3). Wild type N2 *C. elegans,* (Black line), *C. remanei,* (Blue line), **H)** *Caenorhabditis remanei* shows normal sensitivity to 2-nonanone in the absence of food suggesting normal 2-nonanone repellent sensitivity (n=3). Y-axis=Mean avoidance Index (AI) vs X-axis=Time (minutes). Mean ± SEM, Student’s *t-t*est, * p ≤ 0.05, ** p ≤ 0.01, *** p ≤ 0.001. For all assays, n=number of days tested, n.s=not statistically significant when comparing wild type N2 *C. elegans* to other *Caenorhabditis* strains tested in this study. All worms were examined using the same method when in the case of changing food types (*Bacillus megaterium*) or varying volatile chemicals used (2-nonanone (100%)/Benzaldehyde (100%). All *Caenorhabditis* species were examined in comparison to wild type N2 *C. elegans* as a control in identical behavioral assay conditions.

## Description

An environment is often represented by numerous sensory cues. In order to better survive, an animal often needs to detect and process sensory cues simultaneously to make an appropriate behavioral decision. The *Caenorhabditis elegans* (*C. elegans*) genome encodes homologues of a significant number of molecules expressed in mammalian brains, which allows characterizing of the molecular and circuit basis for multi-sensory behavior during decision-making (Bargmann, 1998). In addition, studies have demonstrated various genes and neurons in behavioral differences previously observed in sensory behavior and decision-making when comparing different organisms, such as, nematodes. These variations in behavior involve differences in neuronal signals and neurons. For example, catecholamine signaling, neuropeptide Y-like receptors and pheromone chemoreceptors (Debono and Bargmann, 1998, Srinivasan *et al.*., 2008; Bendesky *et al.*., 2010, Mcgraph *et al.*., 2011). In this study, we investigate the behavioral differences across various species of nematodes, by examining multiple types of *Caenorhabditis* species and how they may differ in a multi-sensory behavioral assay (Harris *et al.*., 2019). The multisensory behavioral assay involves examining how different species of nematodes that are exposed to conflicting cues, behave when compared to the standard wild type, *C. elegans* Bristol N2 worms. We use a multi-sensory behavior paradigm to address these questions to determine any difference in ‘2-nonanone-dependent food leaving’, that assesses food leaving during exposure to the repellent 2-nonanone. Wild type N2 *C. elegans* were examined as the control in comparison to other *Caenorhabditis* species, including, *Caenorhabditis remanei* (*C. remanei*)*, Caenorhabditis nouraguensis (C. nouraguensis),* and *Caenorhabditis portoensis (C. portoensis)*.

Wild type N2 *C. elegans* in the lab are able to leave *E. coli* OP50 food lawns efficiently when exposed to undiluted 2-nonanone repellent (Harris *et al.*., 2019, Fig. 1A-B). In each experiment, we compared each nematode species to wild type *C. elegans* Bristol N2 (*Caenorhabditis* Genetics Center, CGC). A *Caenorhabditis* species*, C. nouraguensis* (*Caenorabditis sp.17)* left an *E.coli* OP50 food lawn during exposure to 2-nonanone similar to wild type N2 *C. elegans,* suggesting *C. nouraguensis* performs 2-nonanone-dependent food leaving with the same food leaving dynamics as wild type *C. elegans* N2 strain (Fig. 1B, Felix MA *et al.*., 2014). Interestingly, we then identified a species that left the food lawn significantly slower than wild type N2 *C. elegans*. The nematode, *C. portoensis* left the *E. coli* OP50 food lawn significantly slower during exposure to 2-nonanone (Fig. 1C). This nematode showed normal movement to the edge of the food lawn furthest away from the 2-nonanone, but remained at the edge of the food lawn for a significantly longer period without leaving the food (Fig. 1C, Felix MA, 2014). In addition to finding nematode species that show delayed food leaving compared to wild type N2 *C. elegans*, we also identified a nematode species that showed enhanced 2-nonanone-dependent food leaving. The nematode species, *C. remanei*, left *E. coli* OP50 food much faster during exposure to 2-nonanone (Fig. 1D, Sudhaus **et al.*,*1974). We also confirmed that for the enhanced food leaving phenotype seen in *C. remanei* was not due to enhancement in sensitivity to 2-nonanone, using the standard 2-nonanone avoidance assay. We found that *C. remanei* showed no enhanced avoidance to 2-nonanone across a time course of 60 minutes (15, 30, 45, 60) (Troemel *et al.*., 1997, Fig. 1H). This suggests that nematodes, such as, *C. remanei* mayexhibit differences in food leaving during repellent exposure due to potentially different response to food signals from the food lawn, during the decision to leave food and not due to differences in sensitivity to the 2-nonanone repellent that drives food leaving. It will be interesting to examine whether delayed food leaving observed in *C. portoensis* is related to differences in 2-nonanone sensitivity*.*

In addition, we have identified that the delayed and enhanced food leaving seen in *C. portoensis* and *C. remanei*, respectively, during exposure to 2-nonanone was also evident when exposing these two nematode species to a second odor repellent, undiluted benzaldehyde, that at high concentrations is also repulsive and drives food leaving in wild type N2 *C. elegans* (Fig. 1E and 1F, Harris *et al.*., 2019, Troemel **et al.*,* 1995; Luo *et al.*., 2008). Suggesting, the delayed and enhanced food leaving differences observed are general to multiple repellents. Finally, we examined changing food types during the assay to determine if we observed similar 2-nonanone-dependent food leaving behavior using different food patches. We show that using a *Bacillus megaterium* food patch instead of *E. coli* OP50 patch did not affect the enhanced food leaving in *C. remanei*, suggesting this faster food leaving behavior during exposure to 2-nonanone is general to multiple food types (Fig. 1G). Overall, we show an experiment that establishes variation between nematodes when measuring decision-making to leave a food patch during responses to conflicting cues, such as in this example, food and a repulsive cue, 2-nonanone. This provides an avenue to further examine differences in nematode behavior when exposing the worms to complex sets of attractive and repulsive cues.

## Methods

All worms were cultivated under the standard conditions (Brenner *et al.*., 1974). Wild type (N2) hermaphrodites were used in this experiment. Wild type and other *Caenorhabditis* species were prepared in the presence of food (*E. coli* OP50) and staged to use young adult worms for all behavior assays.

**Multi-sensory Behavioral Assay:**

Wild type worms (N2) and all other strains in this study were assayed as previously described (Harris *et al.*., 2019). For all worms, *E.coli* OP50 was used as the food source. For preparation of assay plates, the NGM (Nematode Growth Medium) agar was made and poured into small assay plates (6 cm) and then allowed to cool and solidify (using standard NGM plate preparation protocol). After two days, a liquid suspension of *E. coli* OP50 was prepared; 40 mL of NGM media and *E. coli* OP50 colonies were added to the NGM media, this media was then placed at 26°C overnight. The next morning, the NGM media (*E. coli* OP50 culture) was centrifuged at 3500 rpm for approximately 15 minutes. 35 mL of the NGM was removed and discarded. The *E. coli* OP50 pellet was then re-suspended in 5 mL of NGM through mixing on a table top vortex and then 55 µL of this *E. coli* OP50 culture was then added to the center of an NGM agar assay plate (as seen in Fig. 1A, Schematic of behavioral assay). All worms were grown at 20-23°C on NGM plates that contained a lawn of *E. coli* OP50. For each assay, a population of young adult worms, 25-30 wild type (N2) and different nematodes were placed on separate NGM plates on the bacterial food source (*E. coli,* 55 µL). 2-nonanone (100%, 1µl) was then placed approximately two worm lengths from the food source. All nematodes examined were recorded every 5 minutes to determine how many worms have left the *E. coli* OP50food lawn, until completion at 45 minutes. Food leaving during 2-nonanone exposure was defined as the number of worms that left the food lawn (not present on the food at each 5-minute time point across 45 minutes). For all benzaldehyde experiments, assays were performed in identical conditions to 2-nonanone-dependent food leaving assays. This shows the number of worms that have left the *E. coli* OP50food lawn over 45 minutes. Assays with *Bacillus megaterium* food lawnsused identical assay conditions and worm rearing, and wild type N2 *C. elegans* were used as a control for each condition tested. For data analysis, we analyzed the Mean ± SEM, Student’s *t-t*est, * p ≤ 0.05, **p ≤ 0.01, ***p ≤ 0.001.

**2-nonanone avoidance assay**

To examine the avoidance of 2-nonanone, chemotaxis assays were performed essentially as previously described (Troemel *et al.*., 1997). Briefly, animals were placed in the center of a square plate that was divided into sectors A, B, C, D, E and F and 2 drops of 1 µl of undiluted 2-nonanone was added to one side and 2 drops of 1 µl ethanol was added to the opposite side of the plate as a control. Approximately 100 worms were used in each assay. 2-nonanone avoidance was analyzed by counting the number of worms in the sectors A-B, C-D, and E-F with E-F being furthest away from the 2-nonanone point sources. The avoidance index was calculated as the number of animals in sectors A and B (at 2-nonanone) minus the number of animals in the sectors E and F (at control) and normalized with the total number of animals in all 6 sectors on plate (Avoidance Index between 0 and -1.0). For all data analysis, a student’s t test was performed when comparing wild type N2 *C. elegans* hermaphrodite worms to any *Caenorhabditis* nematode species tested on the same day in parallel conditions. Mean ± SEM, Student’s *t-t*est, * p ≤ 0.05, p ≤ 0.01**, p ≤ 0.001***

## Reagents

**Chemicals used in this study**

All chemicals used in this study, including, 2-nonanone (CasRN: 821-55-6) and benzaldehyde (CasRN: 100-52-7) were purchased from Sigma Aldrich.

**List of worm strains and bacterial strains used in these experiments**

1) N2 *Caenorhabditis elegans* Bristol wild type worms were purchased from CGC

2) *E.coli* OP50 strain purchased from CGC

3) DA1880 *Bacillus megaterium* purchased fromCGC

4) PB219 *Caenorhabditis remanei* was purchased from CGC

5) EG4788 *Caenorhabditis portoensis* was purchased from CGC

6) JU1825 *Caenorhabditis nouraguensis* was purchased from CGC

All strains were provided by the CGC(Caenorhabditis Genetics Center) at the University of Minnesota, which is funded by NIH Office of Research Infrastructure Programs (P40 OD010440).
